# A Patient with Double-Negative VGKC, Peripheral Nerve Hyperexcitability, and Central Nervous System Symptoms: A Postinfectious Autoimmune Disease

**DOI:** 10.1155/2020/3579419

**Published:** 2020-07-29

**Authors:** Birte Eikeland

**Affiliations:** Primary Health Clinic, A. Bergs vei 31, 5089 Bergen, Norway

## Abstract

Research in the last few years has indicated that most voltage-gated potassium channel- (VGKC-) complex antibodies without leucine-rich glioma-inactivated protein 1 or contactin-associated protein-like 2 antibody specificity lack pathogenic potential and are not clear markers for autoimmune inflammation. Here we report on a patient with double-negative VGKC who developed severe peripheral nerve hyperexcitability, central nervous system symptoms with agitation and insomnia, dysautonomia, and systemic symptoms with weight loss, itch, and skin lesions. The disease started acutely one month after an episode of enteroviral pericarditis and responded well to immunotherapy. The patient is presumed to have developed a postinfectious immunotherapy-responsive autoimmune disease. In the setting of anti-VGKC positivity, it seems likely that anti-VGKC contributed to the pathogenesis of the patient's symptoms of nerve hyperexcitability and that the disease was caused by an acquired autoimmune effect on the neuronal kinetics of VGKC. It is still unknown whether or not there are unidentified extracellular molecular targets within the VGKC-complex, i.e., a novel surface antigen and a pathogenic antibody that can cause affected individuals to develop a peripheral nerve hyperexcitability syndrome. This case highlights the fact that less well-characterized autoimmune central and peripheral nervous system syndromes may have infectious triggers.

## 1. Introduction

Peripheral nerve hyperexcitability (PNH) is the term used to describe a group of disorders characterized clinically by muscle cramps, muscle twitching (fasciculations or myokymia), muscle stiffness, and pseudomyotonia (delayed muscle relaxation after contraction). Some patients also experience paresthesias and numbness, implying sensory nerve involvement. PNH includes a spectrum of rare disorders, ranging from the dramatic presentation of neuromyotonia or Morvan syndrome, i.e., coexistence of PNH, dysautonomia, and central nervous system (CNS) symptoms, to more benign variants such as cramp-fasciculation syndrome. The etiology can be genetic, particularly mutations in the voltage-gated potassium channel (VGKC), focal nerve injury, demyelination, toxin-induced, or autoimmune. The PNH syndromes were first associated with VGKC-complex antibodies in 1995 [1]. Initially, these autoantibodies were thought to target epitopes of the VGKC, thereby affecting neuronal excitation and causing spontaneous repetitive discharges arising at or near the motor nerve terminal. However, it is now recognized that most of these autoantibodies target proteins that are linked to the VGKC as a functional complex. These proteins include leucine-rich glioma-inactivated 1 protein (LGI1), which is thought to be restricted to the CNS, and contactin-associated protein-like 2 (Caspr2), which is expressed in both the peripheral nervous system and the CNS.

Furthermore, there is now evidence showing that a substantial proportion of patients with VGKC-complex immunoglobulin G seropositivity are double-negative, i.e., lack specificity for LGI1 and Caspr2 IgG, suggesting that other molecular targets of the VGKC-complex remain to be discovered [2]. However, recent research has found that almost all serum samples from double-negative patients show no binding to the surface of live neurons in culture, implying an absence of pathogenic neuronal surface antibodies [3]. Therefore, the clinical significance of isolated anti-VGKC-complex antibodies has been questioned. It is now thought that VGKC positivity in the absence of antibodies to LGI1 and Caspr2 is not a clear marker for autoimmune neuronal inflammation [4]. Here we report on a double-negative patient with severe peripheral nerve hyperexcitability and CNS symptoms who appears to have developed an autoimmune disease for which immunotherapy was effective.

## 2. Case Report

A 42-year-old Norwegian woman presented in November 2006 with a low-grade fever and severe left-sided chest wall pain that was felt to be pericardial in nature. She was otherwise in good health apart from Raynaud's phenomenon and seronegative Sjögren's syndrome. Her erythrocyte sedimentation rate and C-reactive protein were slightly elevated and her enterovirus antibody titer was positive at 128 (normal, <20). She was diagnosed with viral pericarditis.

One month later, she developed generalized body pain that was accompanied by fasciculations, painful cramps, burning sensations, and hyperalgesia that was so severe that she could not wear a watch, bra, or tight-fitting clothes. She reported painful swallowing and marked skin discoloration was noted on the extensor surfaces of the knees and elbows. There was no joint swelling. She then developed proximal muscle weakness in the thighs and became bed-bound. Blood tests for antinuclear antibody (ANA) and its subgroups were negative. The erythrocyte sedimentation rate and C-reactive protein and creatine kinase levels were normal. No muscle biopsy, neuroimmunology blood tests, or neurophysiological evaluation were performed at that time. She was treated with prednisone 40 mg and weaned over approximately 6 months but without significant benefit. The diagnosis was an immunological reaction with muscle involvement triggered by a viral infection.

The patient slowly improved over a period of 3 years. However, in the autumn of 2009, she started to develop further symptoms, including weight loss, anxiety, difficulty lifting her arms, and inability to drive a car. In the spring of 2010, she developed a tonic-dystonic posture and had difficulty relaxing her shoulders. She also developed severe insomnia and facial flushing with rosacea. Her insomnia was treated with a benzodiazepine that triggered a psychotic reaction during which she became manic, could not sleep, was prone to frequent bouts of singing, and was easily distracted. Eventually, this led to a psychiatric hospitalization during which she was diagnosed to have a somatoform disorder.

**Figure 1:**
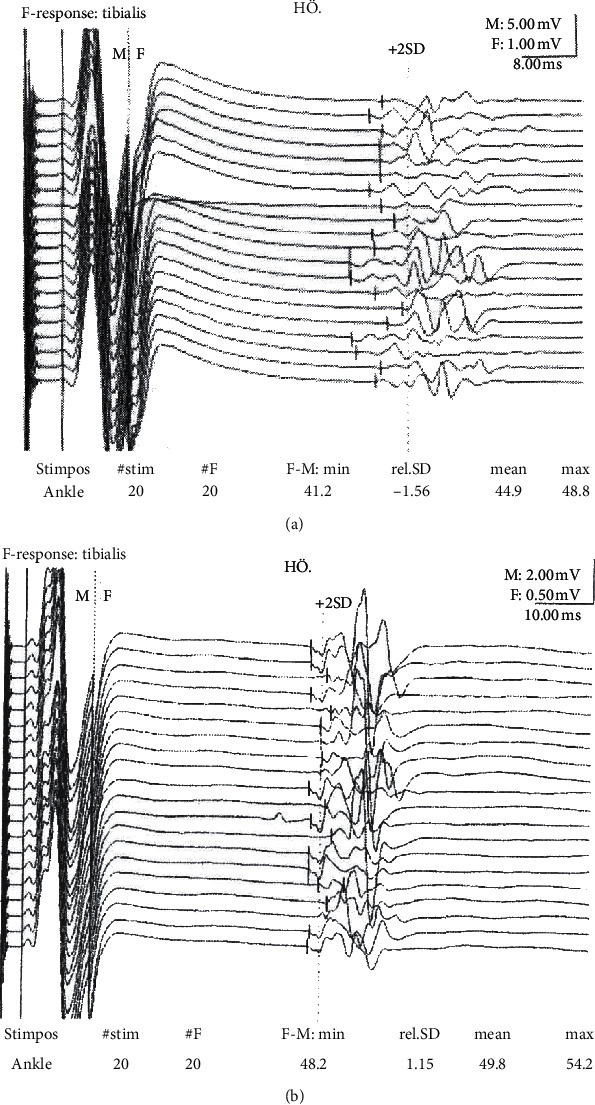
Nerve conduction studies in 2010 (a) indicating F-wave hyperexcitability in the tibial nerve with reduced minimal latency of 41.2 ms, a polyphasic appearance, and persistence. Nerve conduction studies in 2015 (b) showing a normalized minimal latency of 48.2 ms.

The psychotic reaction lasted for 6 weeks and was followed by a severe somatic exacerbation. During the course of a week, she developed torticollis-like head movements, myoclonic-like jerks in her right arm and leg, fluctuating flexion contractures in her elbows, dyspnea caused by both episodes of laryngospasm and chest muscle stiffness, and purplish skin discoloration on her hands and feet. During the subsequent weeks, she developed total body stiffness, palmar flexion contractures of her wrists and fingers, and ankles and toes that were fixed in plantar flexion. She was admitted to the neurology department but underwent limited testing. Needle electromyography (EMG) was essentially normal. Nerve conduction studies indicated F-wave hyperexcitability in the tibial nerve (see Figure 1) but were misinterpreted as normal. Repetitive nerve stimulation was not performed to look for afterdischarges. However, she did have positive ANA and ribonucleoprotein results with nonspecific inflammation and some atrophy in a biopsy from the right deltoid muscle. Unfortunately, she was suspected of malingering and her symptoms were considered to be psychogenic. Therefore, she was again diagnosed to have a somatoform disorder.

Over the next couple of years, she was essentially bed-bound because of her generalized muscle stiffness and contractures. Her symptoms gradually resolved spontaneously, and by 2014, she could walk 3-4 km. However, excessive physical activity at this time triggered a new relapse, which resulted in debilitating disease and the patient becoming bedridden again for about a year.

Her VGKC antibody titer was first measured (by radioimmunoassay, Euroimmun AG, Lübeck, Germany) in May 2015 and found to be positive at 100 pmol/l. She continued to have positive ANA and ribonucleoprotein tests. She was positive for SS-B at this time but later became negative. Other investigations included a comprehensive paraneoplastic evaluation and tests for antiglutamate, anti-GABA, glycine receptor antibodies, Caspr2, and LGI1, all of which were negative. An MRI scan of the brain and an analysis of cerebrospinal fluid were normal. In December 2015, she was readmitted to the neurology department as an inpatient because of her ongoing symptoms. She was presumed to have an autoimmune disorder and started on intravenous immunoglobulin (IVIg) 2 g/kg administered at a daily dosage of 30 g for 5 days, for a total dose of 150 g. She responded well to this treatment, which was repeated at the same dosage in March 2016 and June 2016.

**Figure 2:**
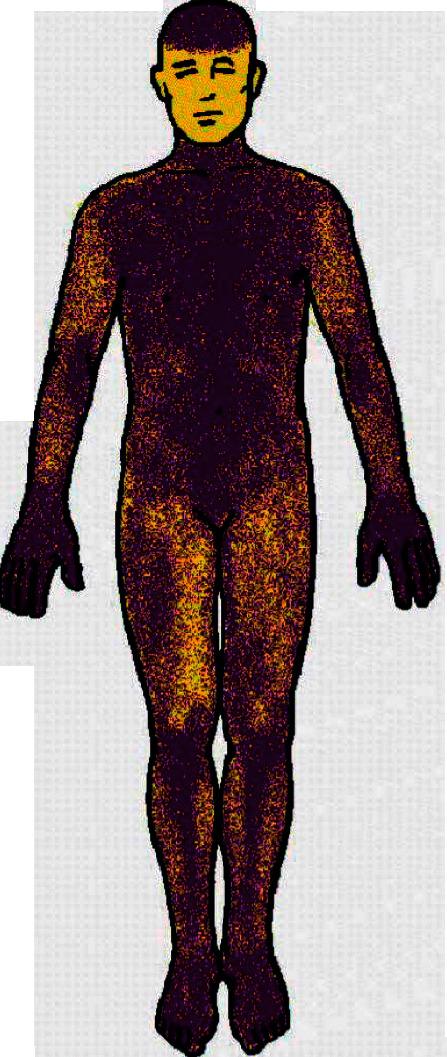
Thermoregulatory Sweat Test. Skin areas in yellow indicate reduced or absent sweat output consistent with small fiber neuropathy.

In July 2016, she was referred to a well-regarded clinic in the US where nerve conduction studies confirmed normal sensory and motor conduction responses. Needle EMG showed slightly larger motor units in the distal muscles, which raised the question of motor involvement, but no neuromyotonia, cramps, fasciculations, or myokymic discharges were seen. An autonomic reflex screen was normal, including the Quantitative Sudomotor Axon Reflex Test. The Thermoregulatory Sweat Test revealed sweating abnormalities with hypohidrosis in the areas of worst pain, specifically the lateral aspects of the legs, thighs, and arms, consistent with small fiber neuropathy (see Figure 2). Laboratory investigations with markers for diffuse autoimmunity revealed a ribonucleoprotein level of >8, which was positive. On paraneoplastic peri-inflammatory evaluation, her anti-VGKC value was 0.01 nmol/l, which was within the normal range. This was 4 weeks after treatment with 150 g of IVIg and likely reflected the good response to treatment. LGI1 and Caspr2 were negative and there was mild immunofluorescent cerebellar staining. The diagnosis was immunotherapy-responsive VGKC-associated autoimmunity with multiple immune-inflammatory markers.

**Figure 3:**
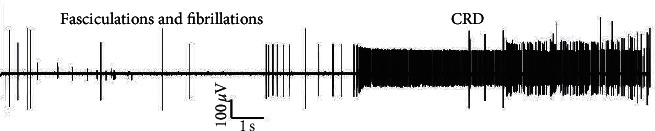
Needle electromyogram showing spontaneous activity in the right abductor pollicis brevis muscle with fasciculations, fibrillations, and complex repetitive discharge at 90–120 Hz.

Further ancillary tests were performed. Serum tested weakly positive for staining live-cell hippocampal neurons in one test at the Nuffield Department of Clinical Neurosciences (Oxford, UK). Immunohistochemistry of brain tissue showed fine granular to smooth fluorescence of the hippocampus and the molecular layer on rat cerebellum 1 : 100 and monkey cerebellum 1 : 32 (Euroimmun AG). Antibodies to subunits of the VGKC channel itself (anti-KCNA 1, 2, and 6) were negative, as was contactin 2 (Euroimmun AG). Paranodal/nodal testing with Caspr1, contactin 1, neurofascin 155, and neurofascin 186 was also negative (Euroimmun AG). Excitability tests using the QTRAC threshold-tracking software were normal. However, needle EMG revealed abnormal spontaneous activity in the form of fibrillation potentials, complex repetitive discharges, and myotonic discharges in the abductor pollicis brevis and extensor digitorum brevis muscles (Figure 3).

Treatment with IVIg was continued after she was correctly diagnosed in 2016. At first, 30 g of IVIg was administered every fourth week (0. 1 g/kg/week). However, her VGKC titer increased again from 10 pmol/l to 98 pmol/l. Therefore, the doses of IVIg were increased to 60–90 g every fourth week (0.2–0.3 g/kg/week). Serial anti-VGKC titers were ≤100 pmol/l but were never again found to be as low as 10 pmol/l. IVIg has been continued in this patient and she now seems to be dependent on this immunotherapy. Although she is still symptomatic, her autoimmune disease is now considered to be in remission. Her functioning and quality of life have improved markedly, given the previous severity of her symptoms and the lengthy course of the illness. She has much less pain and stiffness and a higher exercise tolerance, which has allowed her to resume her daily activities, including ambulation and part-time work. Her response to treatment is very similar to that in another report on a patient who developed VGKC autoimmunity (negative for both Caspr2 and LGI1) after exposure to aerosolized porcine neural tissue [5].

## 3. Discussion

This patient presented with a complex constellation of nerve hyperexcitability symptoms over a period of 10 years before she was diagnosed to have VGKC-associated autoimmune syndrome and treatment with IVIg was initiated. Several of her symptoms are reportedly rare in patients with this type of autoimmune channelopathy, i.e., respiratory distress caused by both laryngospasm and chest muscle stiffness, generalized persistent muscle contractions resulting in stiffness and an extended body posture, generalized pain of neuropathic character, painful swallowing, and facial flushing with rosacea.

Her symptoms were similar to those found in Morvan syndrome, with co-occurrence of CNS symptoms, peripheral nerve hyperexcitability, and autonomic dysfunction. She also had several of the core symptoms [6] described in the clinical spectrum of Caspr2 antibody-associated disease but did not present with Caspr2 or LGI1 positivity. However, Irani et al. [7] reported that 3 of 29 patients with Morvan syndrome did not have Caspr2 or LGI1 positivity and suggested that some patients may have antibodies against other VGKC-complex (or uncomplexed) antigens. There are no clear triggers for Morvan syndrome [8], although a series of 4 patients described in India in 2017 suggested a possible viral trigger [9], as suspected in our patient.

The symptoms and signs in this patient could also be consistent with a diagnosis of autoimmune pain, which is rare and typically presents with additional neurological manifestations [10]. Chronic pain is also a syndromic manifestation of VGKC-complex autoimmunity, and hyperexcitability of nociceptive pathways is implicated [11]. There is a significant association between Caspr2 and pain, but the antigenic VGKC-complex molecule has not been identified in most patients. However, VGKC-complex antibodies are not often found in complex regional pain syndrome [11], despite the occurrence of both pain and autonomic disturbance in patients with the disorder. Our patient presented with a severe pain syndrome and autonomic dysfunction with blue feet, so complex regional pain syndrome could have been a differential diagnosis.

Stiff person syndrome is another diagnosis to consider in this case. Manifestations of this autoimmune movement disorder are truncal and limb stiffness with superimposed spasms. Our patient presented with both limb and total body stiffness and respiratory problems caused by chest muscle stiffness. Nevertheless, most patients with Stiff person syndrome have autoantibodies specific for GAD65 and also some for glycine receptor antibodies. Our patient tested negative for both of these types of autoantibodies.

This patient's disease started acutely after an episode of enteroviral pericarditis. The initial muscle symptoms with severe pain, proximal weakness in the lower limbs, and inflammation-related redness of the skin over the extensor surfaces of the knees and elbows could have suggested a viral myositis. Later she was found to be positive for ANA and ribonucleoprotein. This nonneurological autoimmunity, together with the premorbid history of Raynaud's phenomenon and seronegative Sjögren's syndrome, suggests a possible overlap with a connective tissue disease and cross-disease autoantibodies. Furthermore, our patient was identified as belonging to a group of patients with VGKC who are not positive for LGI1 and Caspr2 but tend to have multiple other immune-inflammatory markers. It is likely that immunotherapy-responsive VGKC-IgG-positive patients without LGI1 and Caspr2 autoantibodies have either coexisting or other immune-inflammatory processes without known primary autoimmunity targets.

Our patient's first positive VGKC antibody result in May 2015 was 100 pmol/l, which is a low titer. Hart et al. [12] found no correlation between the antibody titer and the severity of the clinical features in patients with peripheral nerve hyperexcitability. However, her anti-VGKC titer was not measured in 2010 when she presented with debilitating disease and she was diagnosed incorrectly to have a somatoform disorder. Given the rarity and phenotypic heterogeneity of this autoimmune channelopathy, our patient's case is an example of how such atypical neurological presentations can be dismissed as a functional disorder [13]. As a consequence, immunotherapy with IVIg was delayed for 10 years. This raises the questions of whether or not severe disease could have been avoided and if earlier immunotherapy would have resulted in a full clinical recovery in this patient.

Almost all her relapses and exacerbations were triggered by physical activity, as reported by Hart et al. [12], who found that exercise triggered all motor features of peripheral nerve hyperexcitability in most of their patients. Myokymia/fasciculations in the foot and fasciculations in the knee and finger provided clinical evidence of peripheral motor nerve hyperexcitability in our patient, as illustrated in the video clips in Supplementary Materials (video S1 showing fasciculations in the finger, video S2 showing myokymia/fasciculations in the foot, and video S3 showing fasciculations in the knee).

Needle EMG recordings in 2019 revealed abnormal spontaneous activity suggestive of hyperexcitability. Earlier EMG studies did not show any spontaneous discharges. However, Hart et al. [12] found that the extent of the electrophysiological abnormality did not relate to the clinical severity of muscle hyperactivity, and a single EMG study is liable to spatial as well as temporal sampling error. Nerve conduction studies performed in 2010 showed reduced minimal F-wave latency in the tibial nerve suggestive of hyperexcitability (see Figure 1). In 2004, Nobrega et al. [14] investigated the F-waves of 100 healthy individuals to establish normative data for clinical use. Their minimal latency value for the tibial nerve was 47.0 ± 4.1 ms, and our patient's minimal latency was reduced to 41.2 ms. In 2015, when our patient was clinically better, nerve conduction studies were repeated and the minimal latency for the tibial nerve was normal at 48.2 ms. Hyperexcitability features are often found in the EMG when F-waves are abnormal; in this patient, only one F-wave showed hyperexcitability and the EMG was more or less normal (with only slightly larger motor units and some units having a polyphasic appearance). F-wave hyperexcitability can be seen in the presence of continuous muscle activity, as in peripheral nerve hyperexcitability/neuromyotonia, but there are very few case reports in the literature documenting this phenomenon [15, 16].

Excitability testing with the threshold-tracking software QTRAC did not reveal any pathological findings, but the distal portion of the motor nerve is not investigated with threshold tracking. The likely locus for the generation of spontaneous discharges in patients with autoimmune peripheral nerve hyperexcitability is at the motor nerve terminal or intramuscular arborization area [12, 17]. The motor nerve terminal is relatively unprotected by the blood-nerve barrier and is potentially more vulnerable to antibody-mediated autoimmune attack.

The Thermoregulatory Sweat Test revealed sudomotor abnormalities with generalized hypohidrosis suggestive of a small fiber neuropathy (see Figure 2) and the Quantitative Sudomotor Axon Reflex Test was negative. These findings are compatible with a preganglionic sudomotor lesion. VGKCs also localize to the presynaptic nerve terminals, and the pathophysiology of peripheral nerve hyperexcitability is dysfunction of VGKCs. Other manifestations of autonomic dysfunction were the patient's facial flushing with rosacea and blue discoloration of the feet, most likely as a result of cutaneous dysautonomia. Hyperhidrosis was temporarily present. Dysautonomia is a prominent feature of Morvan syndrome. This patient's autonomic involvement started at the same time as her CNS symptoms with profound insomnia and agitation, which are very similar to the cardinal symptoms found in Morvan syndrome.

The question arises as to why an autoimmune etiology is most likely in this case. The patient had an underlying immunological profile with Raynaud's phenomenon and seronegative Sjögren's syndrome. Importantly, her illness was preceded by enteroviral pericarditis one month before the acute onset of her nerve hyperexcitability symptoms. She subsequently developed nonneurological autoimmunity with positive ANA and ribonucleoprotein, as well as a transiently positive SS-B. In 2010, she presented with both a movement disorder and psychiatric symptoms; a psychiatric presentation together with a movement disorder is a strong indicator of a possible autoimmune component [18]. Serum tested weakly positive for staining live-cell hippocampal neurons, suggesting the possibility of an extracellular pathogenic antibody [19, 20]. Our patient had a good clinical response to IVIg that was accompanied by a reduction in her anti-VGKC titer, which confirms that her disease was genuinely immunotherapy-responsive. Overall, the clinical picture and ancillary tests substantiate an autoimmune etiology for this patient's presenting neurological symptoms.

## 4. Conclusion

This patient presented with a clinical phenotype of severe nerve hyperexcitability symptoms with both CNS and peripheral nervous system involvement. In the setting of anti-VGKC positivity, it seems likely that anti-VGKC contributed to the pathogenesis of her nerve hyperexcitability. This finding is in contrast with recent research, indicating that almost all double-negative VGKC-complex antibodies lack pathogenicity and are not a clear marker for autoimmune inflammation. However, the decision whether or not to treat a patient for a suspected autoimmune disease should be guided by clinical reasoning including ancillary testing. Factors contributing to the autoimmune etiology and diagnosis in this patient were the preceding enteroviral infection, acute/subacute onset of muscle symptoms, psychiatric symptoms presenting at the same time as a movement disorder, positivity of markers for diffuse autoimmunity, positive staining of live hippocampal cells, and a clinical response to IVIg.

## Supplementary Materials

Supplementary Materials Three supplementary video clips (Videos S1–S3) showing myokymia/fasciculations in the foot and fasciculations in the knee and finger accompany this manuscript.
