# Corrigendum to “A Patient with Double-Negative VGKC, Peripheral Nerve Hyperexcitability, and Central Nervous System Symptoms: A Postinfectious Autoimmune Disease”

**DOI:** 10.1155/2020/7359671

**Published:** 2020-10-13

**Authors:** Birte Eikeland

**Affiliations:** Primary Health Clinic, A. Bergs vei 31, Bergen 5089, Norway

In the article titled “A Patient with Double-Negative VGKC, Peripheral Nerve Hyperexcitability, and Central Nervous System Symptoms: A Postinfectious Autoimmune Disease” [1], the authors wish to update the legend of Figure 3 to add the attribution as follows:

Figure 3: Needle electromyogram showing spontaneous activity in the right abductor pollicis brevis muscle with fasciculations, fibrillations, and complex repetitive discharge at 90–120 Hz. Published with permission from “Department of Clinical Neurophysiology, Rigshospitalet, Copenhagen.”
